# To succeed as health care innovators, pharmacists need to cultivate an entrepreneurial mindset

**DOI:** 10.1177/17151635231164622

**Published:** 2023-03-27

**Authors:** Peter Chengming Zhang, Zubin Austin

**Affiliations:** Southlake Regional Health Centre, Newmarket, Ontario; Health Services Research and the Leslie Dan Faculty of Pharmacy, University of Toronto, Toronto, Ontario

Creating new, improved business solutions often requires innovators to pursue a high-risk career path that pushes boundaries, institutions and traditions alike. When this process destroys older, established practices, it is sometimes referred to as creative destruction. However, despite pharmacists having lived through this process throughout the evolution of pharmacy practice, this concept appears nowhere in the traditional pharmacy education curriculum.

For pharmacists, this means that the profession risks stagnation despite continued advancements in technology. In areas where pharmacy has advanced, many of these cases have been driven by innovators outside of the profession. For example, the use of computer software to support pharmacy workflow has evolved tremendously in the past 2 decades and has been largely driven by the entrepreneurial achievements of nonpharmacists.

The “Kroll Pharmacy Management Solution” is one such case. Developed 40 years ago, Kroll had worked to eventually become the market leader when it came to helping pharmacies go paperless. Rather than a pharmacist, it was Tony Kroll, a computer science student turned businessman, who was able to identify an opportunity to improve the pharmacist workflow (personal communication, 2023).

There were 2 major competitors that already occupied this space: ProPharm, which was built by pharmacists, and Zadall, which was built by computer programmers. But Kroll was able to surpass both these products in market share by balancing design and implementation, and his success in this effort was driven by his background in computer science and his investment in deeply understanding his customers: pharmacists.

Pharmacists already have a good understanding of pharmacy. What is missing are the other pieces of the puzzle, for example, the ability to identify opportunity, experience with risk management when trying something new and being open-minded to developing skills outside of an established expertise. Much like how Kroll had to explore pharmacy outside of his computer science education, pharmacists may actually be better positioned to become successful entrepreneurs when they develop skills that are not directly related to their profession.

Kroll stated that “motivation” is likely the most important contributing factor to his success. Regardless of the drive, whether it is financial, career satisfaction or something else, a passion to innovate is essential to bringing new ideas into the market. For pharmacist innovators, while this passion exists, with a number of successful pharmacist-led companies such as MedEssist, Cubic Health and MedMe clearly demonstrating this, the current pharmacy education curriculum does not prepare them for a path in entrepreneurship.

A failure to innovate within the profession carries a number of risks. One risk is that pharmacists become dependent on external agents in order to improve their internal processes. This dependency may result in outside interests superseding pharmacist interests or obligations. Another risk is that pharmacists fail to capture opportunities in areas that they are, in fact, best suited to lead, such as innovations in medication adherence and safety. These risks suggest that fostering an entrepreneurial mindset is essential to advancing the profession to the best interest of its practitioners.

## But what is an entrepreneurial mindset?

The Network for Teaching Entrepreneurship (NFTE) published a paper in 2019^1^ to help better understand the domains that are represented in the entrepreneurial mindset. These include opportunity recognition, future orientation, flexibility and adaptability, critical thinking and problem solving, creativity and innovation, communication and collaboration, initiative and self-reliance and risk management.

These are domains that have been previously demonstrated by pharmacist entrepreneurs. In a recent article in the *Canadian Pharmacists Journal*, “Travel Health Pharmacy: A New Model for Sustainability,”^2^ a travel pharmacy as a new practice setting was identified as an opportunity for pharmacist-led care. A political, economic, social and technological (PEST) analysis was conducted to identify opportunity. Furthermore, a strengths, weakness, opportunities, threats (SWOT) analysis was conducted alongside a market sizing exercise to demonstrate the need for critical thinking required to assess business viability.

Although skills such as conducting a PEST or SWOT analysis can be taught didactically, a number of domains recognized by NFTE are not necessarily developed in a classroom setting. For example, risk management is best developed through practical learning experiences.

Uniquely, pharmacists take on additional risk through the opportunity costs of pursuing entrepreneurship in place of a stable and financially rewarding full-time clinical career. In addition, students who intend to pursue pharmacy may be attracted to the stable nature of the profession, and the risks of founding a company can be perceived as unattractive.

For pharmacists, the need to foster an entrepreneurial mind-set is essential to the profession. In a time when technologies such as machine learning and pharmacogenomics have the potential to cause critical disruptions to the profession, pharmacists need to be the ones to develop market solutions that will allow them to own and shape the future of health care.
